# A Reversibly Sealed, Easy Access, Modular (SEAM) Microfluidic Architecture to Establish *In Vitro* Tissue Interfaces

**DOI:** 10.1371/journal.pone.0156341

**Published:** 2016-05-26

**Authors:** Vinay V. Abhyankar, Meiye Wu, Chung-Yan Koh, Anson V. Hatch

**Affiliations:** Department of Biotechnology and Bioengineering, Sandia National Laboratories, Livermore, California, 94551, United States of America; University of Illinois at Chicago, UNITED STATES

## Abstract

Microfluidic barrier tissue models have emerged as advanced *in vitro* tools to explore interactions with external stimuli such as drug candidates, pathogens, or toxins. However, the procedures required to establish and maintain these systems can be challenging to implement for end users, particularly those without significant in-house engineering expertise. Here we present a module-based approach that provides an easy-to-use workflow to establish, maintain, and analyze microscale tissue constructs. Our approach begins with a removable culture insert that is magnetically coupled, decoupled, and transferred between standalone, prefabricated microfluidic modules for simplified cell seeding, culture, and downstream analysis. The modular approach allows several options for perfusion including standard syringe pumps or integration with a self-contained gravity-fed module for simple cell maintenance. As proof of concept, we establish a culture of primary human microvascular endothelial cells (HMVEC) and report combined surface protein imaging and gene expression after controlled apical stimulation with the bacterial endotoxin lipopolysaccharide (LPS). We also demonstrate the feasibility of incorporating hydrated biomaterial interfaces into the microfluidic architecture by integrating an ultra-thin (< 1 **μ**m), self-assembled hyaluronic acid/peptide amphiphile culture membrane with brain-specific Young’s modulus (~ 1kPa). To highlight the importance of including biomimetic interfaces into microscale models we report multi-tiered readouts from primary rat cortical cells cultured on the self-assembled membrane and compare a panel of mRNA targets with primary brain tissue signatures. We anticipate that the modular approach and simplified operational workflows presented here will enable a wide range of research groups to incorporate microfluidic barrier tissue models into their work.

## Introduction

Microfluidic barrier tissues (MBTs) are advanced *in vitro* culture models that combine microengineering techniques with living cells to help address the need for biologically representative assays to explore complex biological interactions [1,2]. Typical MBTs are monolithic polymer structures composed of synthetic microporous culture membranes irreversibly sealed between elastomeric microfluidic channel networks; the individually addressable channels above and below the suspended culture membrane provide excellent control over biophysical/biochemical microenvironments and facilitate development of polarized co-cultured cell populations within the architecture. Recently, microfluidic approaches have been used to achieve barrier models of the vasculature, lung, blood-brain barrier, gut, kidney, and liver [3]. These systems extend the capabilities of conventional barrier models, such as the Transwell assay, and open new avenues for exploration by enabling quantitative cell and tissue level readouts including viability; metabolic activity; toxicity; barrier permeability; cell migration; and surface protein expression from within defined culture environments. [4–7]. Even though MBTs have provided important biological insights, they can be difficult to implement in laboratories without in-house engineering expertise. In particular, processes associated with effective cell seeding, culture maintenance, and access to cells for downstream analysis can be challenging. The aim of the work presented here is to introduce a new approach that incorporates specialized pre-fabricated microfluidic modules to help streamline and simplify experimental workflows.

MBTs require effective bonding between elastomeric channels and suspended culture membranes to achieve defined apical and basolateral culture environments. Common bonding techniques employ adhesive glues or oxygen plasma treatments to prepare surfaces for permanent attachment. Although highly effective in polymer-to-polymer attachment, biological culture membranes are difficult to incorporate because of challenges related to the formation of leak-proof seals between polymeric microfluidic channels and hydrated materials [8,9]. Permanently sealed architectures also present potential challenges for effective cell introduction onto the culture membrane surface, and can require significant fluidic manipulation and handling to collect cells and lysate from the system for downstream analysis. Several groups have presented alternative microchannel attachment techniques, including external vacuum/magnetic manifolds or mechanical attachment schemes, to compress fluidic channels against growing cell layers or planar substrates to create seals in hydrated environments [10]. However, these large form factor approaches can be expensive to produce for disposable operation and can require the use of complex peripheral equipment.

With the mapping of the human genome and advancements in bioinformatics, genomics has emerged as a powerful approach to explore the activities that occur upstream of protein expression [11]. For example, gene readouts can identify whether a drug candidate can engage targeted signaling pathways with specificity, or guide the development of a medical countermeasure by mechanistically exploring host-pathogen interactions [12]. Even though nucleic acid isolation protocols are well established within conventional lab-on-a-chip microfluidic platforms [13,14], the robust RNA isolation required for analysis is difficult to achieve with current MBT platforms. The challenge likely stems from the inevitable sample losses that occur along the fluidic path during lysate collection resulting from dead volumes and transport along high surface-area-to-volume ratio channels [15]. Alternative RNA isolation techniques with reduced sample handling steps could significantly simplify nucleic acid collection and help provide easy access to gene level readouts from membrane-based microfluidic systems.

There is a significant effort in the microfluidic community to create easy-to-use systems to improve adoption by life science researchers by limiting the need for external pumps and other instrumentation [16], [17]. Here we focus on developing a user-friendly workflow to reduce experimental complexity while introducing new capabilities. Our Scalable, Easy-Access, Modular (SEAM) platform is composed of pre-fabricated microfluidic modules that enable i) precise cell seeding; ii) facilitate integration of hydrated biomaterial culture membranes; iii) support robust nucleic acid isolation; and iv) promote multi-tiered experimental readouts. As proof of concept, we use SEAM to validate inflammation related surface protein markers and gene expression readouts from primary human microvascular endothelial (HMVEC) cells after apical stimulation with the bacterial endotoxin lipopolysaccharide (LPS). We also demonstrate SEAM compatibility with hydrated culture interface materials by incorporating a self-assembled suspended membrane with brain-specific Young’s modulus using magnetic latching.

## Materials and Methods

**Fig 1:**
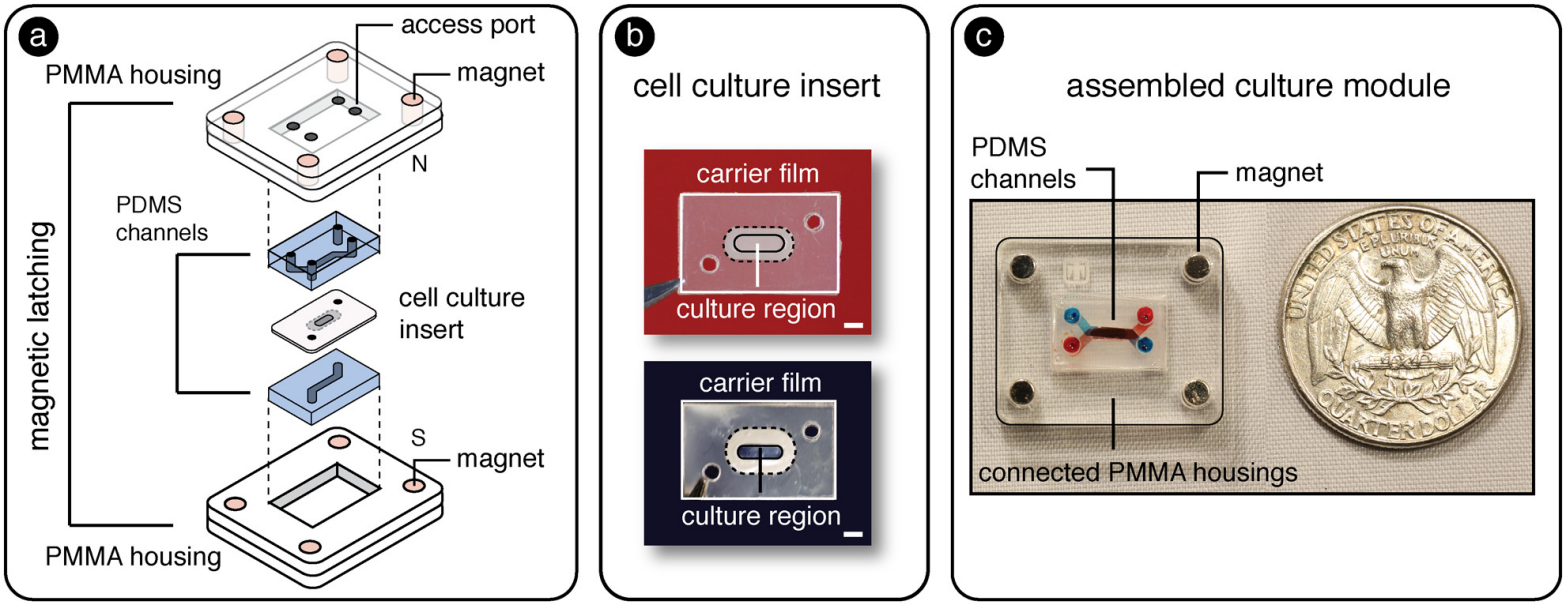
a) Schematic representation of SEAM architecture composed of PMMA housings with embedded magnets, PDMS channels, and a removable cell culture insert. b) Images of the removable culture inserts including porous track etched (top) and soft self-assembled (bottom) culture regions. Scale bar = 1 mm. c) Assembled SEAM culture module with top and bottom microfluidic channels filled with red and blue dyes to demonstrate distinct fluidic compartments within a magnetically sealed architecture.

To achieve a “user friendly” approach to establish and analyze tissue interfaces, we developed the SEAM architecture composed of specialized modules using a laboratory prototyping method combining soft-lithography, laser machining, and lamination. As shown in Fig 1a, the culture module was constructed from i) laser cut PMMA housing layers (PMMA thickness 1.5875 mm (1/16”), McMaster Carr, VersaLaser 40W CO_2_ laser, Universal Laser Systems) with embedded rare earth magnets (K&J Magnetics), ii) molded PDMS microchannels, and iii) a removable cell culture insert.

### PMMA Housings and magnetic latches

Rigid plastic housing layers were constructed by laminating two laser cut pieces of PMMA (base layer: 18 mm x 22 mm, and cavity: 18 mm x 22 mm with 7 mm x 11 mm cutout) using pressure sensitive adhesive sheets (Fralock). Each housing layer contained rare earth magnets (2.54 mm diameter) oriented such that the top and bottom housings had opposite magnetic poles facing one another. The cavities were designed to accept the PDMS containing channels as described below.

### PDMS channel fabrication

Polydimethysiloxane (PDMS, Sylgard 184) microchannels (h = 0.25 mm, w = 1 mm, l = 9 mm) were fabricated using standard soft-lithography replica molding techniques [18]. Briefly, UV reactive photoresist SU-8 100 (MicroChem) was spin coated onto a silicon wafer, soft-baked, and then exposed to UV light through a high-density transparency mask (Fineline Imaging, Inc.) to define channel features. The wafer was then immersed in developer solution (MicroChem) to remove non-crosslinked SU-8 and hard baked to establish a “master” used to create microfluidic channels. To achieve PDMS blocks of defined total height, the master was placed into a mold cavity composed of a PMMA ring (outer diameter = 250 mm, inner diameter = 200 mm) attached to a PMMA disk (diameter = 300 mm) using pressure sensitive adhesive (Fralock). The mold cavity was then filled with degassed PDMS prepolymer (10:1 base to catalyst ratio,). A mylar transparency sheet was placed on top of the filled mold to create a flat upper surface and covered with an additional PMMA ring (diameter = 300 mm). The assembled mold was clamped and placed in an oven for 6 hours at 65°C. The resulting PDMS block (with total height of 1.7 mm) was removed from the mold and cut to size (7 mm x 11 mm) with a razor blade. Access ports were cored using a 1 mm biopsy punch (Miltex). PDMS blocks were inserted into the upper and lower PMMA housings with the channel features oriented toward the interior of the device.

### Cell culture inserts

As shown in Fig 1a, magnetic latching allows culture membranes to be sandwiched in between fluidic channels and sealed in place to support compartmentalized (apical/basolateral) cultures. We demonstrated the use of two cell culture materials, i) a standard track etched micro-porous culture membrane and ii) a hydrated, self-assembled culture membrane to highlight the flexibility of the magnetic sealing approach (see Fig 1b). The hydrated, suspended membrane represents a material that is difficult to incorporate into MBT models due to aforementioned bonding incompatibilities. A polyester track-etched culture membrane (0.4 **μ**m pore diameter, Sterlitech) was laser cut, sandwiched, and laminated between two laser cut polyethylene (PE) sheets containing an open central region and pressure sensitive silicone adhesive on one face (Fralock). The non-porous PE isolated the fluidic compartments and ensured that communication between the channels only occurred through the nanoporous culture membrane. The PE sheets also allowed the culture insert to be easily transferred between specialized modules.

For the culture insert that included a hydrated material, PE sheets and porous membranes (0.1 **μ**m, Steriletch) were laser cut to create open central regions. A nanoporous track etched membrane was arranged so that a small edge extended into the open region. We adapted a liquid-liquid supramolecular self-assembly chemistry pioneered by Stupp [19–21] to establish a water permeable culture membrane that tethered to the carrier insert. A PDMS reservoir was filled with a 2% w/v solution of hyaluronic acid (HA) in acetate buffer (pH 5.7, 50 mM), and a culture insert was positioned on top. A second PDMS reservoir was placed over the insert and filled with a 3% w/v solution of peptide amphiphile (PA) in acetate buffer (pH 5.7, 50 mM). The structure of PA contained a palmitic acid tail, beta sheet forming sequence, and a charged head group (C_16_V_3_A_3_K_3_). HA is a large molecular weight negatively charged linear polymer that is abundant in mammalian extracellular matrix. Structures are shown in S1 Fig. When the solutions came into contact with one another, a solid membrane instantaneously formed at the liquid-liquid interface through a self-assembly process driven by hydrophobic collapse of the palmitic acid tails and charge screening between the positively charged PA heads and negatively charged HA. Membrane thickness could be tuned based on liquid-liquid contact time [22,23].

After self-assembly, membranes were gently washed in PBS (10 mM sodium phosphate, 150 mM sodium chloride, pH 7.4). Membranes were then mounted onto a cleaned glass coverslip in a fluid cell filled with PBS. The Young’s modulus for the membrane was extracted from a plot of cantilever deflection versus displacement by fitting data to a spherical Hertz contact equation using a pre-calibrated AFM cantilever modified with 0.005 mm spherical probe as described by Soofi [24]. Thickness of the membrane was determined by AFM in contact mode using a silicon nitride probe with tip dimension of 27 ± nm and 800 nm height (Novoscan). Data were exported in NanoScope Analysis (Bruker) for analysis in Origin Pro (Origin Labs). The molecular weight cutoff of the membrane was determined by measuring the transport of fluorescently labeled dextrans purchased from Sigma (10 kDa, 40 kDa, 70 kDa, 120 kDa, 150 kDa) across the membrane in 24 hours. Characterization data are shown in S2 Fig.

### Assembly

The attraction forces between the magnets in each housing layer sealed the PDMS channels against the culture insert and established a liquid-tight seal capable of withstanding ~ 35 kPa of applied pressure. An assembled SEAM culture platform using a track etched culture membrane insert is shown in Fig 1c with red dye (top channel) and blue dye (bottom channel) used to identify distinct compartments. Each channel could be separately perfused with cell medium to maintain the culture or introduce an experimental test compound (e.g. drug, virus, or environmental toxin). The magnetic latching mechanism allowed the carrier insert (and attached cells) to be introduced and removed from SEAM modules and handled independently from the fluidic network. As shown in S1 Video the magnetic latching and culture insert sealing process could easily be performed by a three-year-old volunteer.

### Direct seeding module

**Fig 2:**
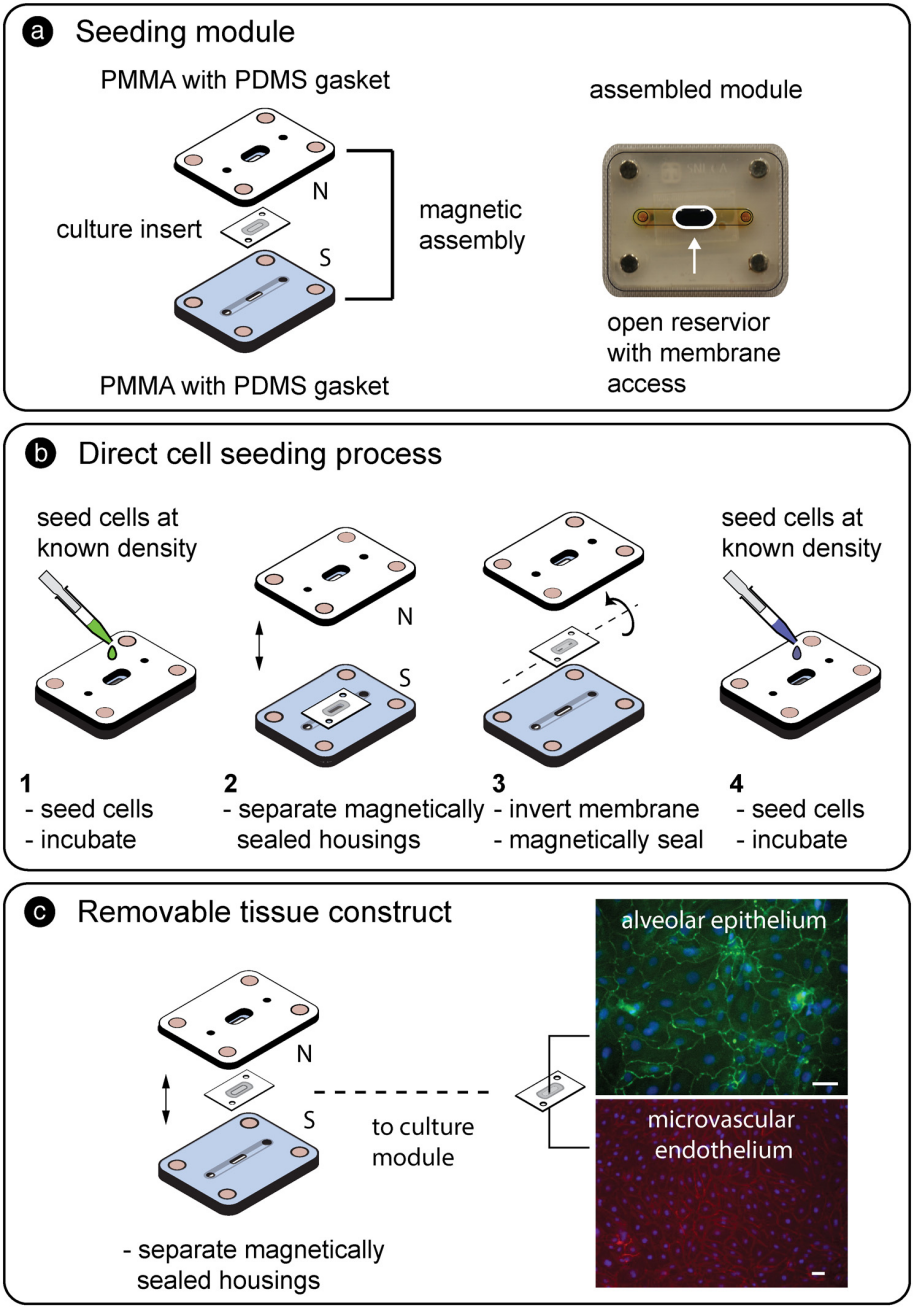
a) Schematic and assembled seeding module composed of top and bottom housing layers and a magnetically sealed culture insert. In the assembled module (right), the bottom channel was filled with yellow dye and the open access cell addition region filled with blue dye to aid visualization. b) Workflow (steps 1–4) to establish co-cultured tissue interfaces. The desired cell number was added to the culture region of the insert and allowed to attach. The insert was then decoupled from the PMMA housings, flipped over, resealed, and seeded with a second population. c) Representative image of co-cultured primary human alveolar epithelium stained for occludin (green) and primary human microvascular endothelium stained for VE-cadherin (red) both with nuclear counterstain (blue). Scale bars = 40 μm.

As shown in Fig 2a, magnetic latching allowed a carrier insert to be reversibly sealed within a two-part PMMA seeding module where cells were directly introduced to the culture region (surface area = 0.05 cm^2^).

The fabrication process utilized the laser cutting and lamination processes described for the culture module. The direct seeding module consisted of a top PMMA housing with embedded magnets, access ports, and a central reservoir. A laser cut PDMS sheet (0.125 mm, Interstate Specialty Products) was attached to the bottom with pressure sensitive adhesive (0.050 mm, Fralock) to promote leak-free seal formation around the culture insert during magnetic compression. The bottom PMMA housing contained magnets, a fluidic channel, and a laser cut PDMS sheet defining fluidic access ports. The culture membrane was aligned onto the bottom layer over the central access port and the housings were magnetically connected. The bottom channel and top reservoir were filled with culture medium to support cell attachment, and the desired number of cells was added directly over the culture region of the carrier insert. The seeding module was incubated for 4–6 hours until cells adhered to the culture membrane. For co-cultures, the magnetically latched PMMA housings were separated and the membrane was flipped and resealed between the housings. The bottom channel was filled with appropriate culture medium to support the attached cell population, and the exposed side of the membrane was then seeded with the second cell population. After cell adhesion, the insert (with attached cells) could then be transferred to the culture module.

### Micro-perfusion module

Although alternative methods are emerging [25], conventional microfluidic approaches rely on syringe pumps to control media perfusion to maintain culture health, provide biomechanical stimulation, and deliver experimental test compounds. External pumps are attractive because of the dynamic control they provide, but equipment costs can be prohibitive and implementation within an incubated environment can be challenging. To provide a user-friendly option for fluid perfusion to support simple cell growth, we utilized a gravity-based perfusion module comprised of horizontally oriented liquid-filled reservoirs separated by a defined height. In typical gravity based approaches with vertically oriented reserviors, the difference in liquid height between free liquid surfaces produces a hydrostatic pressure drop, **Δ**P = **ρ**g**Δ**z, that is reduced as the difference, **Δ**z, between the liquid levels approaches zero. Horizontal reservoirs have been shown [26] to maintain a hydraulic pressure drop across a connected microfluidic channel network because Δz remains constant as discussed below.

**Fig 3:**
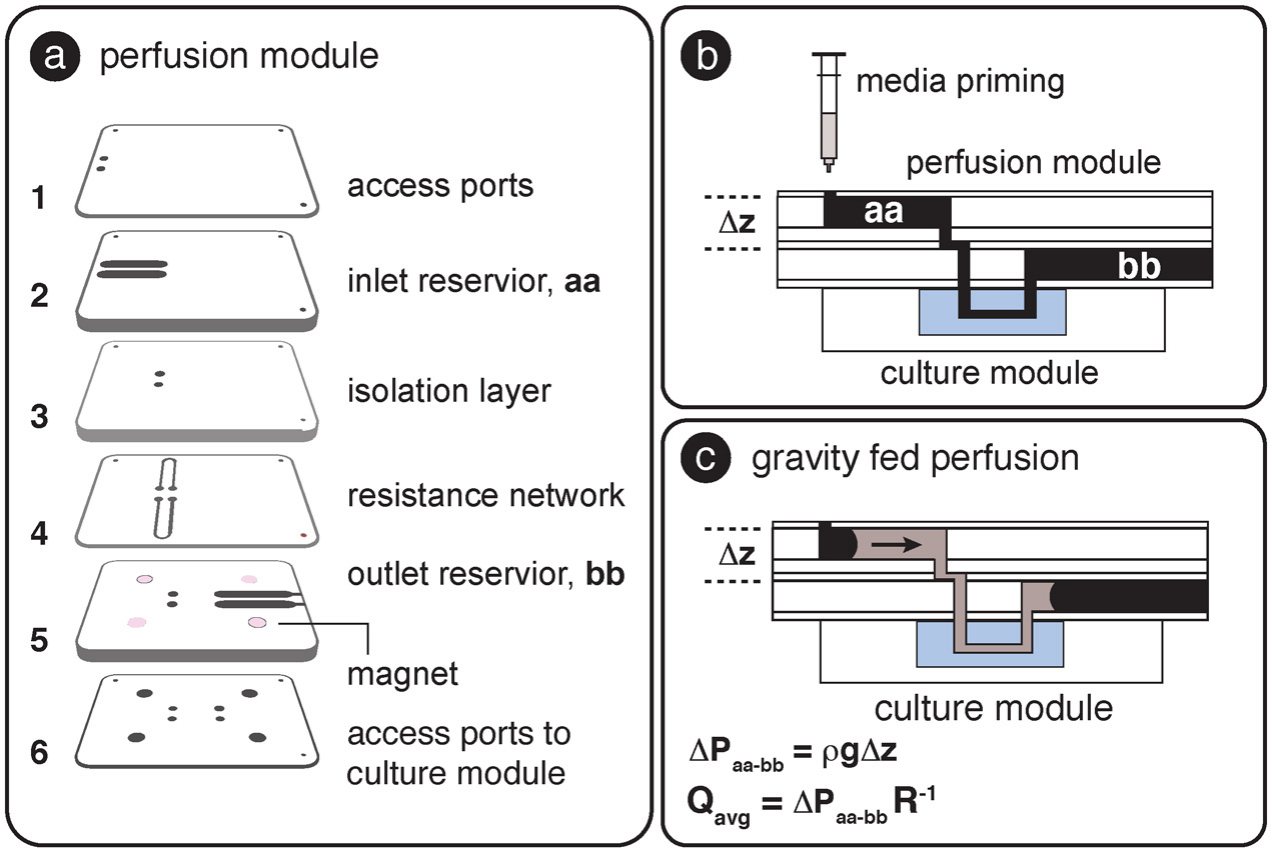
a) Layers comprising the gravity fed perfusion module. b) Side view of connected perfusion and culture modules. The fluidic circuit (inlet reservoir, aa, through the culture module to the outlet reservoir, bb) was gently primed with medium using a syringe. c) Once filled, surface tension kept the medium from spilling out of the reservoirs, and the difference in hydrostatic pressure **Δ**P induced fluid flow from inlet to outlet reservoirs.

The micro-perfusion module was fabricated using the combination of PMMA laser machining, pressure sensitive adhesive, and lamination described previously; the layer-by-layer fabrication approach allowed flexibility in prototype design. As shown in Fig 3a, the layers included horizontally oriented inlet and outlet fluid reservoirs (**layers 2 and 5** respectively with PMMA thickness = 2.5 mm), a fluidic isolation layer that separated the reservoirs (**layer 3**, h = 1 mm), a microfluidic resistance network (**layer 4**, channel dimensions: h = 0.02 mm, w = 0.5 mm, L = 25 mm), ports to access inlet reservoirs (**layer 1**), and fluidic interconnection (**layer 6**) between the perfusion module and the magnetically connected culture module.

As shown in Fig 3b, the connected perfusion and culture modules were magnetically coupled and gently primed with media until the channels were filled and an air-liquid meniscus was established in each reservoir. The surface tension between the medium and the reservoir surfaces was sufficient that the liquid did not spill out in the horizontal configuration and the difference in hydrostatic pressure (**Δ**P_aa-bb_ = **ρ**g**Δ**z) between the inlet and outlet reservoirs induced flow from aa to bb. The meniscus moved horizontally as the liquid flowed through the circuit; since there was no change in the relative heights of the liquid within the reservoirs (**Δ**z), and gravitational acceleration and liquid density were constant, **Δ**P was also constant. Flow contributions related to capillary pressure were assumed to balance because the inlet and out reservoirs were constructed from the same materials and had the same internal dimensions.

With the constant pressure drop (**Δ**P_aa-bb_) and fluidic resistance, R, in low aspect ratio channels defined as [27]:

$$R=\;\frac{12\mu L}{wh^{3}}{\left[1-\frac{192h}{\pi^{5}w}\text{tanh}\left(\frac{\pi w}{2h}\right)\right]}^{-1}$$ (1)

The average flow rate in the network, Q_avg_, was calculated from the expression Q_avg_ = **Δ**P R^-1^ with **μ** = 0.0007 [Pa sec] at 37°C, and w, h, L referring to the width, height and length of each segment in the fluidic path respectively. Table 1 shows the relationship between **Δ**z and the calculated average flow rate in the culture channel. The flow rate in our system (with **Δ**z = 3.62 mm) was calculated to be 2.42 **μ**L hr^-1^, corresponding to 58 **μ**L used per day (one full medium exchange in the culture channel per hour). New perfusion modules were used at the end of each culture period.

**Table 1:** Differential height vs. predicted flow rate and shear stress.

	Differential Height, Δz [mm]				
	1	3.6	5	10	20
Q_avg_ [μL hr^-1^]	1.36	2.42	3.34	6.68	13.6
τ [dyne cm^-2^]	0.00013	0.0045	0.0006	0.0013	0.0026

The corresponding shear stress in the culture channel was calculated to be 0.00045 dyne cm^-2^ using the expression [28] in Eq 2, with γ (aspect ratio of culture channel) = 0.25 [–].

$$\tau =\frac{6\mu Q}{wh^{2}}\left\{\begin{array}{cc} 2.95 & \gamma <0.25\\ 1.158+{\left(1-\left(\gamma \right)\right)}^{-2} & 0.25\leq \;\gamma \leq 0.85 \end{array}\right\}$$ (2)

Shear controlled environments can be beneficial in culture maintenance applications using shear-sensitive cells such as neurons and stem cell populations [29]. Calculated shear stress results are tabulated in Table 1. For studies where larger shear stress values are required, increasing the pressure head (i.e. 15.875 mm or 5/8”), removing the resistance network, and reducing the culture channel width to 0.40 mm and height to 0.1 mm enables theoretical shear stresses on the order of 10 dyne cm^-2^ to be achieved.

In the embodiment shown here, the purpose of the perfusion module was to demonstrate an optional stand-alone option capable of maintaining primary human cells in culture without the need for instrument-driven pumping techniques. However, the modular nature of the SEAM platform allows conventional pumping methods, including syringe pumps, to be implemented if desired (S5 Fig that shows connection to an external pump and interconnection between multiple culture modules).

### Cell seeding

The culture insert was sealed in the seeding module and the bottom channel was filled with appropriate culture medium. The upper reservoir was then loaded with fibronectin (5 **μ**g mL^-1^) and incubated (37°C at 5% CO_2_) overnight to coat the membrane. The reservoir and channel were emptied via pipette, washed with PBS (10 mM sodium phosphate, 150 mM sodium chloride, pH 7.4) and filled with appropriate culture medium (ScienCell). Primary HMVEC (ScienCell) were thawed according to manufacturer’s instructions and plated onto fibronectin-coated tissue culture plates. After one subculture, cells were trypsinized and resuspended at a concentration of 500,000 cells mL^-1^. 2 **μ**L of the suspension was added to each open well (~1000 cells input) of the seeding platform directly over the culture region of the sandwiched culture insert. The cells were visualized with an inverted microscope (Olympus IX-70) to ensure uniform coverage. The seeding module was then incubated until cells adhered to the membrane (4–6 hours). Rat cortical neurons (ScienCell) were handled according to manufacturer’s instructions. Because of the fragile nature of the neurons, cells were seeded directly into a HA coated polystyrene tissue culture plate (Nunc) or onto a self-assembled membrane at a density of 30,000 cells cm^-2^.

For co-culture, HMVEC cells were seeded as described above. The membrane was then decoupled and flipped over. The bottom channel was filled with HMVEC medium and the seeding reservoir was filled with human pulmonary alveolar epithelial cell (HPAEpiC) culture medium (ScienCell). HPAEpiC were then added to the reservoir at a concentration of 20,000 cells cm^-2^. Medium was changed twice daily. After three days in culture, the cells on the membrane were fixed and permeabilized for immunocytochemistry.

### Immunocytochemistry

Cells were fixed with 4% paraformaldehyde (Sigma) in PBS for 20 minutes followed by permeabilization with 0.1% Triton X-100 in PBS for 15 minutes. Cells were then blocked with 2% BSA in PBS for 2 hours. Primary antibodies against MAP2 (1:000, Sigma) or ICAM-1 (1:40, R&D Systems), Occludin (1:1000, Alexa 488 conjugated, Invitrogen), or vascular endothelial cadherin (VE-Cad, 1:10, R&D Systems) were introduced and incubated for 6–8 hours at 4°C. The primary antibody solution was removed and cells were gently rinsed with PBS and then incubated for two hours with secondary antibodies conjugated to Alexa 647 (1:500, Life Technologies), or Qdot 625 (1:500, Life technologies). Nuclei were stained with DAPI (Life Technologies) at 15 **μ**g mL^-1^. 12-bit images were collected through ImagePro software on an Olympus IX-70 microscope with a Photometrics CoolSnap HQ2 camera. Exposure settings were chosen to minimize background fluorescence. Image processing was performed in ImageJ and settings were applied identically to all samples. Membranes were imaged directly in the device or removed and mounted onto microscope slides, covered with PBS, and sealed with a coverslip prior to imaging.

### Gene analysis

*Rat cortical neurons*: Culture inserts were decoupled from the culture module with sterile forceps and placed in 150 **μ**L of RNAzol RT (Molecular Research Center). From this point on, the RNA extraction proceeded according to manufacturer’s instructions. Cells were lysed via pipetting and the solution was certrifuged at 12,000g for 2 minutes. The supernatant was placed in an RNase-free tube and 150 **μ**L of absolute ethanol was added to the solution. The mixture was vortexed, then placed on a Zymo-Spin IIC column, and centrifuged at 12,000xg for one minute. The column was then washed and treated with DNase to remove any contaminant DNA. RNA was eluted in 50 **μ**L of ultrapure water. RNA quality was determined using the RNA 6000 kit on the Bioanalyzer (Agilent Technologies). For cDNA synthesis, RNA was then reverse transcribed to cDNA using the iScript cDNA Synthesis kit (Bio-Rad) according to manufacturer’s specifications. Each reaction was carried out in duplicate wells with housekeeping genes (GAPDH [5’ VIC], ACTB [5’ FAM], RPL13A [5’ TET]) detected in multiplex using TaqMan probes (Applied Biosystems endogenous control sets, Life Technologies) in one well and the gene of interest detected in the other well using SYBR Green PCR master mix (Life Technologies) on a CFX96 (Bio-Rad). Gene targets included: Neurodap1, NCAM1, c-fos, trkA, (forward and reverse primers shown in S3 Fig). The **ΔΔ**Ct method was used to calculate mRNA quantities relative to housekeeping genes after experimental well factor normalization. For each biological replicate, two reverse transcription reactions were performed with one control without RT. For each cDNA generated, three technical replicates were performed with two controls without template. Determination of threshold Ct at 99% confidence was determined by multiplying the standard deviation of the NTC for each target gene by 6.965. Control experiments using RNA from normal rat brain (Takara Clontech) were treated as above to generate data for comparison. Data represent average with standard deviation.

*HMVEC*: After the LPS stimulation, RNA extraction of the cells in the device was performed using the Quick RNA Microprep kit from Zymo (Cat. R1050). The culture inserts (and cell layer) were decoupled from the culture modules and immediately submerged in 600 **μ**L of ZR buffer from the Microprep kit and centrifuged to lyse all cells. From this point on, the RNA extraction proceeded according to manufacturer’s specifications. The RNA was eluted with 10 **μ**L of nuclease free water, and RNA quality was determined using the Agilent RNA 6000 Pico Kit (Agilent, 5067–1513). For RNA extraction from intact devices, an equivalent volume (600 **μ**L) of lysis buffer from the RNA Microprep kit was manually pipetted into the culture module and collected. The collected solution was then loaded onto the purification columns, and extraction proceeded as directed by the manufacturer.

For cDNA synthesis, 5 ng of RNA from each sample was used to generate cDNA using the Superscript VILO cDNA synthesis kit (Life Technologies, Cat. 11754-050), using manufacturer’s recommended conditions. For qPCR, 100ng of cDNA was used in individual Taqman gene expression assays (Applied Biosystems). The relative expression of von Willebrand factor (Hs00169795_m1), ICAM-1 (Hs00164932_m1), Cadherin 5 (Hs00901463_m1), CXCL1 (Hs00236937_m1), CXCL2 (Hs00601975_m1), and CCL2 (Hs00234140_m1) were analyzed using the **ΔΔ**Ct method, with GAPDH (Hs99999905_m1) serving as positive control for normalization, and the expression level without LPS stimulation served as basal level. For each cDNA generated, three technical replicate were performed with two controls without template. Determination of threshold Ct at 99% confidence was determined by multiplying the standard deviation of the NTC for each target gene by 6.965.

## Results

**Fig 4:**
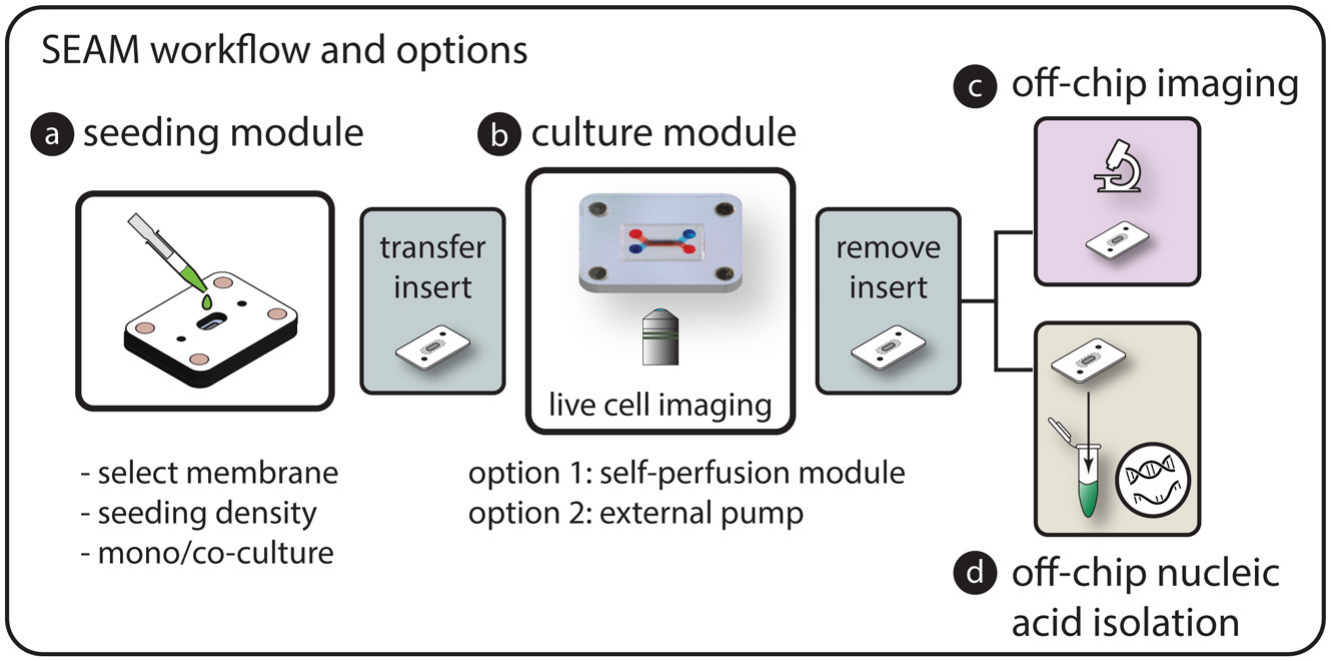
SEAM experimental workflow options. a) Cells are seeded directly on the culture region of the desired insert at a known density using the seeding module. b) The culture insert with attached cells is transferred to the culture platform for long-term perfusion culture and exposure studies. c) The insert can then be directly transferred for off chip lysis and nucleic acid isolation or d) mounted on a slide for imaging. Cells can also be imaged within the platform using a long working distance objective. e) The modular approach enables a combination of imaging and gene level responses to be easily explored.

The modular approach and removable culture insert in the SEAM platform enabled workflows that combined the direct cell access provided by open cultures with the characteristic culture capabilities found in microfluidic systems. As described schematically in the workflow (Fig 4), culture inserts were reversibly coupled to seeding modules for precise microscale seeding, then transferred to the microfluidic culture module for cell culture and LPS stimulation studies using the micro-perfusion module (option 1). Following stimulation, experimental readouts were obtained from differential immunocytochemistry of surface proteins or by decoupling the culture insert from the module and transferring it to a commercial off-chip workflow to achieve high quality nucleic acid isolation for downstream analysis.

### Validation of SEAM readouts using HMVEC stimulated with LPS

**Fig 5:**
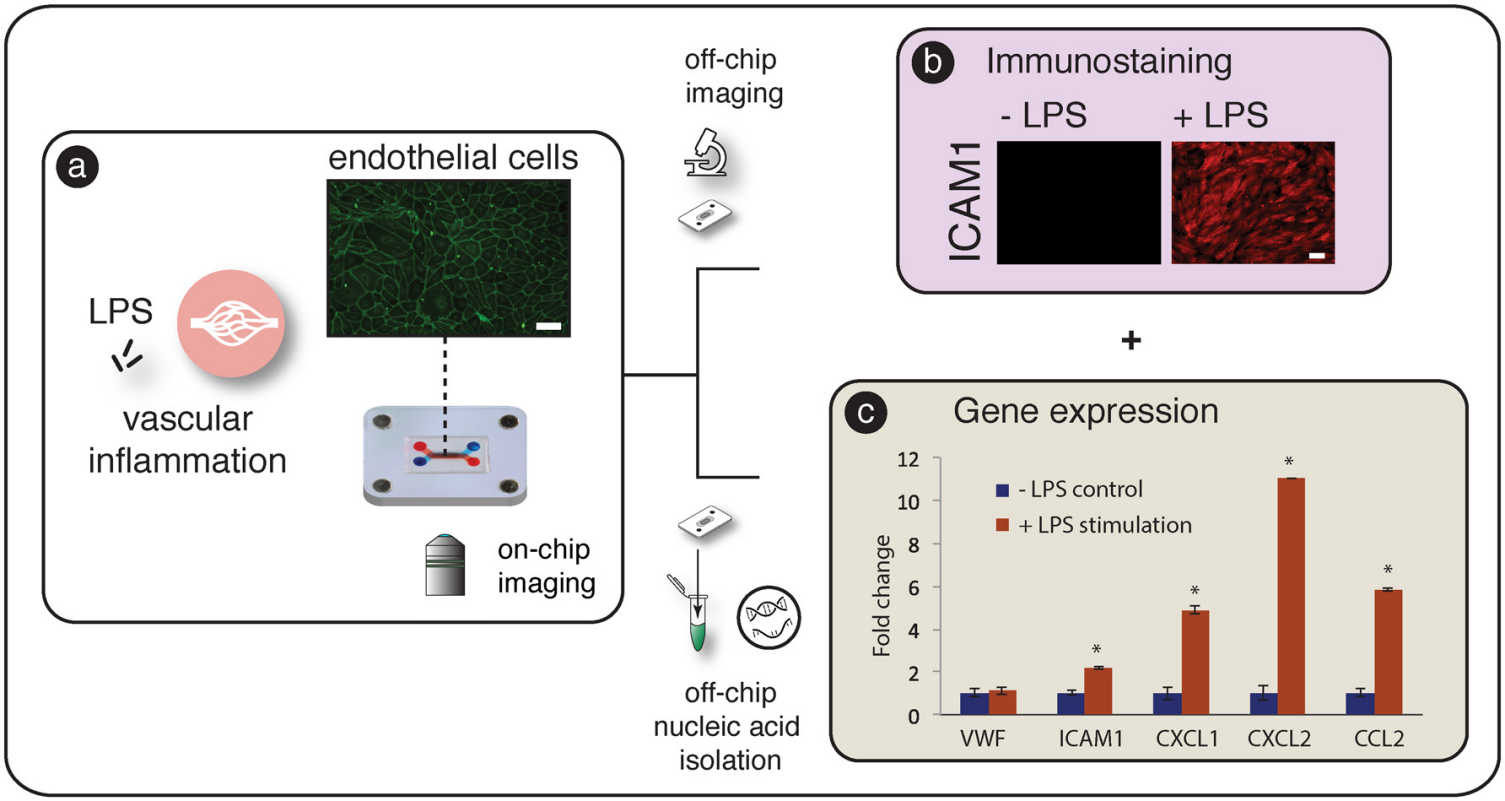
SEAM readouts after LPS stimulation. a) To explore SEAM readouts that mimic vascular inflammation, human microvasular endothelial cells were seeded and transferred to the coupled culture and micro-perfusion modules. b) After apical simulation with LPS for 4 hours, cells were and stained in-channel and the membrane was transferred to a glass slide to explore ICAM1 surface expression after exposure to stimulation. Scale bar = 40 μm. c) To demonstrate rapid and robust RNA isolation, inserts were removed from the architecture and transferred to off-chip RNA isolation. Statistically significant (p<0.001) differences in mRNA expression were observed in ICAM1, CXCL1, CXCL2, and CCL2 with VFW (endothelial marker) remaining constant. SEAM is able to replicate both gene expression and differential surface protein responses to LPS stimulation with an easy to use workflow.

HMVEC line the blood vessels and play a critical role in mediating vascular permeability, regulation of blood flow, wound healing, and inflammation [30]. The bacterial endotoxin lipopolysaccharide (LPS) is a cell wall component of gram-negative bacteria, and is frequently used to study inflammation. LPS is known to activate Toll-like receptor 4 (TLR4) receptors on endothelial cells and induce up-regulation of cell surface adhesion molecules to facilitate neutrophil arrest [31] and increase the production of chemokines to recruit and activate dendritic cells and macrophages [32]. We used this well-studied model for vascular inflammation to validate multi-tiered SEAM readouts as shown in Fig 5a. Primary HMVEC were seeded onto the microporous culture region of the insert (surface area = 0.05 cm^2^) using the direct seeding module. The culture surface area of the membrane was selected to accommodate a monolayer of contact inhibited cells (~20,000 cells cm-^2^) using an input population of 1000 cells. 1000 cells could be easily and reproducibly pipetted and helped to reduce per/assay cost. The size of the carrier membrane (7 mm x 11 mm) was designed to fit directly into the cell isolation vial of a standard RNA isolation protocol with having to be cut or otherwise manipulated.

After cell attachment, the culture insert was transferred to the culture module and connected to the micro-perfusion module for 24 hours of culture (Fig 5a). The perfusion module was then replaced with a new module, and cells were exposed to LPS through the apical channel for 4 hours while the basal channel was perfused with culture medium. After stimulation, devices were randomized and selected for ICAM-1 immunocytochemistry or RNA extraction for downstream gene expression analysis. Decoupling and transferring the culture insert (with attached cells) allowed RNA to be isolated from the entire cell population using an optimized commercial off-chip technique without requiring fluidic sample handling. We successfully achieved high quality RNA isolation (RIN ~ 10, S4 Fig) from a small initial cell population. Cells isolated using in-channel techniques also showed high RIN (Average = 9.5) but exhibited 2x lower RNA yield (34.1 ± 1.38 ng vs. 17.3 ± 1.17 ng). Although further purification steps could counteract low RNA yield, the membrane transfer method significantly simplified the process and reduced potential sources of error by eliminating sample handling steps. Direct transfer of a cell population provides an avenue for cost effective experimental scale up and supports whole-genome analysis techniques such as RNA-Seq or miRNA-Seq that explore global transcriptomic profiles without *a priori* target specification.

Fig 5b and 5c show cells stained for ICAM-1 and the gene expression profile reflecting LPS induced mRNA changes. Significant increases of mRNA expression were found for ICAM-1, CXCL1, CXCL2, and CCL2 (* *p* < 0.001). ICAM-1, a surface adhesion molecule involved in the arrest of rolling leukocytes, was upregulated 2.5 fold while CXCL1 and CXCL2, chemokines involved in the recruitment of neutrophils and lymphocytes, increased 5 fold and 11 fold respectively. CCL2 has been shown to promote dendritic cell and monocyte infiltration and mRNA expression increased 6 fold compared to unstimulated control. mRNA levels of the endothelial phenotypic marker Von Willebrand Factor remained unchanged as expected for healthy control. Staining of ICAM-1 between the stimulated and unstimulated cells demonstrated that mRNA changes were translated to protein level changes in the system. Using the self-contained SEAM platform, were able to demonstrate mRNA and protein level changes resulting from controlled LPS stimulation.

### Neuronal culture and comparison of gene expression on different substrates

The overall goal of microscale *in vitro* barrier models is to establish physiologically representative culture environments (e.g. biochemical cues, mechanical stimulation, cell-cell communication, and cell-matrix interactions) in which to study tissue level responses. The impact of biochemical cues and mechanical stimulation within microfluidic architectures has been well documented in the literature [33]. It is also well established that biophysical interactions between cells and their substrate can affect gene expression and drive phenotypic changes through outside-in signaling [34–37]. Even though there has been significant progress in the development of biomaterials and tissue scaffolds with well-defined biomimetic properties [38,39], techniques for suspending these culture interfaces within microfluidic platforms has been slow to emerge. The reversibly sealed architecture provided by SEAM can overcome this issue and enable the facile incorporation of hydrated culture interfaces to help mimic the structure of the target tissue.

For example, the cerebral cortex or “grey matter” plays an important role in several high level processes including movement and perception. The brain ECM has a unique composition with low percentages of fibrous proteins (e.g. collagen and fibronectin), and high percentages proteoglycans including HA [40,41]. To demonstrate SEAM capabilities incorporating biomimetic membranes and isolating nucleic acids, we quantified gene level differences between rat cortical cells grown on a soft suspended HA/PA biomimetic interface with brain specific Young’s modulus (~ 1kPa) to those grown on a coated rigid plastic surface.

**Fig 6:**
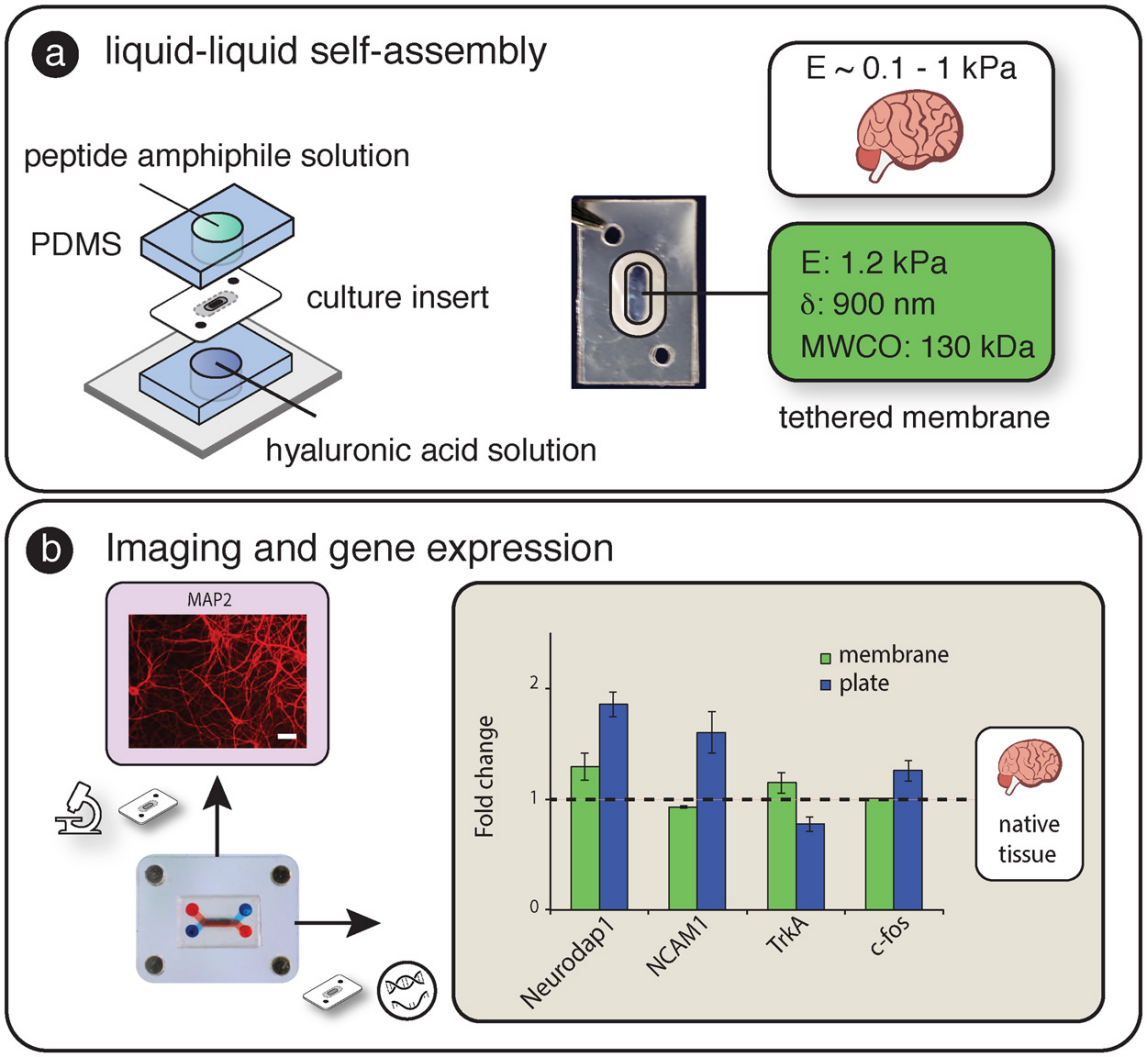
a) Incorporation of self-assembled membrane into laminated carrier insert. Young’s modulus of membrane is similar to brain tissue. b) After 3 days in culture on the SA membrane, separate membranes were stained for MAP2 (dendridic marker, scale bar = 40 μm) and processed for gene expression analysis. Gene expression of Neurodap1, NCAM1, TrkA, and c-fos, on the soft self-assembled substrate were closer to rat brain gene expression (dashed horizontal line) than those cultured on rigid substrates.

As shown in Fig 6a, the “soft” HA/PA membrane (E ~1 kPa) was tethered to the carrier insert using a liquid-liquid self-assembly process. After seeding and three days in culture on the self-assembled membrane (within the culture module) or standard tissue culture plate, neurons were either stained for the dendritic marker, MAP2, or used in gene expression analysis for NeuroDap 1, which plays a role in synaptic communication [42]; neuronal adhesion molecule 1 (NCAM-1), a glycoprotein involved in cell–cell adhesion, neurite outgrowth, and plasticity [43]; c-fos, an indirect marker of neuronal activity [44]; and tropomyosin receptor kinase A (TrkA), a high affinity receptor for nerve growth factor (NGF) that activates transcription factors to control signaling in soma [45,46]. These mRNA targets represent a broad cross-section of adhesion proteins, receptors, and neuronal activity metrics and were chosen to help assess the potential importance of substrate stiffness from several perspectives. When compared with neurons plated on rigid tissue culture plastic substrates, cells cultured on the self-assembled culture membrane showed interesting similarities to gene expression profiles from RNA isolated from rat brain tissue (Fig 6b). The inclusion of hydrated biomaterials using the SEAM architecture combined with robust RNA isolation provides experimental capabilities that can help to improve the relevance of future *in vitro* microfluidic barrier models by enabling experimental control over outside-in signaling.

## Discussion

To support user-friendly methods to establish, maintain, and analyze microscale tissue interfaces, we developed a self-contained platform that consisted of a removable cell culture insert and distinct prefabricated modules for microfluidic operations. The reversibly sealed SEAM architecture allows the culture insert to be rapidly coupled, decoupled and transferred between modules to achieve an efficient experimental workflow. The modular also approach provides unique advantages related to i) efficient cell seeding, ii) the simple inclusion of tailored biological interfaces, iiii) robust nucleic acid isolation, iv) generation of multi-tiered readouts, and v) simplified user-friendly operation.

### Cell culture insert

The concept of the removable cell culture insert is a key aspect of the SEAM platform. The insert consisted of a micro-scale porous cell culture region surrounded by a non-porous carrier that facilitated transfer and coupling with different fluidic modules. The lamination of laser cut PE sheets (with pressure sensitive adhesive on one face) is compatible with commercially available micro and nanoporous membranes that have been optimized to provide low auto-fluorescence, high optical clarity, and defined pore size distributions. Materials such as ultrathin nano-membranes [47,48] collagen films [49], hydrogel sheets [50], patterned substrates [51,52], commercial EMC products [53], and layered composite biomaterials [54] can also be included to further tune the culture interface to reflect the physiology of the tissue.

Conventional microfluidic techniques to permanently seal membranes between elastomeric channels primarily use oxygen plasma bonding and have difficulties forming robust, liquid-tight seals when hydrated materials are present. With the magnetic latching and removable culture insert concept, the culture materials do not have to be directly compatible with the overall fabrication method of the architecture. We demonstrated a tethering approach to incorporate a thin, self-assembled membrane with Young’s modulus tailored to mimic the brain environment (~ 1 kPa). This interface-based liquid-liquid membrane self-assembly fabrication technique [55] was selected because it is difficult to incorporate this type of culture substrate into a membrane-based microfluidic platform. The inclusion of hydrophilic (polyester) and hydrated membrane materials (HA/PA) also enabled simple wetting and filling without surface modification and limited bubble introduction and formation within the channels. In the unlikely case that a bubble is observed, the system can be disassembled to remove to obstruction.

Our adaptation of the self-assembled membrane into the culture insert enables handling and manipulation of the suspended 900 nanometer thin membrane throughout the workflow (e.g. seeding, culture, imaging, and nucleic acid isolation). Thin culture membranes can help accurately represent the dynamics of cell-cell communication across a tissue interface. For example, paracrine signaling factors traveling across a 10 μm thick culture membrane requires 100x longer than with a 900 nm thick membrane. Several barrier tissues (including the alveolar-capillary interface [56] and blood-brain-barrier [57]) contain polarized cell layers separated by sub-micrometer distances; the ability to include tailored ultra-thin biomaterials provides a unique capability to replicate physiological tissue architectures. We observed this phenomenon in our proof of principle demonstration exploring gene level changes in rat neurons cultured on soft and rigid interfaces. The functional implications of these gene level changes must be explored in more detail, and SEAM architecture provides a convenient approach to incorporate advanced biological interfaces to enhance the relevance of *in vitro* assays.

We found that decoupling and transferring the culture insert enabled high quality nucleic acid isolation from small cell samples. After the experimental exposure, the insert (with the entire cell population) was decoupled from the architecture using reversible magnetic latching and transferred directly to the workflow of the off-chip isolation protocol. The ability to move an entire cell population without manipulation helped limit sample losses that inevitably occur when transporting cell lysates within high surface-area-to-volume ratio microfluidic channels. Using the transfer approach, we were able to achieve RIN values approaching the maximum value of 10 from a ~1000 cell input population. RIN values were also high using in-channel lysis and collection (Average = 9.5) but resulted in ~ 2x less RNA and collection compared with culture insert transfer method. The removable culture insert technique allowed the entire cell population to be simply and rapidly transferred for RNA isolation without requiring fluid manipulation.

Although we demonstrated differential gene expression readouts in this work, transfer of the insert enables a wide variety of optimized commercial techniques including analysis to explore DNA damage, genotoxicity, and whole genome transcriptomics (e.g. miRNA-Seq or RNA-Seq) that explore global pathway activation patterns that result from interaction with a drug candidate or pathogen. Even though it is well recognized that the activation of a signaling pathway at the mRNA level does not necessarily lead to protein expression and functional response, gene level readouts provide important insights to identify potentially interconnected pathways that can be used to assess potential toxicity and guide therapeutic development. qPCR-based protein assays (e.g. TaqMan Protein Assays) can be included to the workflow to directly correlate mRNA expression with protein levels and explore post-translational protein modification. In addition, the removable culture insert can be mounted onto a glass slide for high-resolution imaging of intra-cellular and surface-level protein localization. The transferable insert concept opens the door for advanced multi-tiered readouts that provide unique insights into how a tissue interacts with an external agent.

### Direct Cell seeding

In permanently sealed membrane-based systems, tissue interfaces are established by introducing cells to the interior of the device by flowing a cell suspension through the microfluidic channels that are in contact with the culture membrane; flow is stopped, and cells settle onto the membrane surface. The sedimentation process along the entire channel length makes it difficult to control initial cell density and reproducibly introduce the same number of cells to the system. Special care must also be taken to prevent the introduction of bubbles during the flow based cell introduction process. Cell seeding is significantly easier in conventional open format culture platforms, such as Petri dishes and multi-well plates, where cells are directly added to the culture surface with a pipette tip. The simplicity of the open seeding approach is replicated here by reversibly coupling a culture insert within a seeding module; a defined cell number is directly added to the culture region through the open reservoir. Direct seeding can significantly reduce the number of cells that are needed per experiment and enable the use of rare or expensive cell populations including stem cells, primary human cells, and clinical relevant samples.

For example, a typical microfluidic system is seeded with ~ 20,000 primary human cells on one side of the membrane [58]. For a commercial primary human cell population costing ~ $600 per million cells, and assuming 100% of cells are delivered uniformly to the culture region without sample loss, the conservative cell-related cost is $12/assay. With the direct seeding technique, ~ 1000 cells can be introduced directly to a defined culture region with minimal sample loss since fluidic manipulation is not required. The corresponding cell-related cost is decreased to $0.60/assay (20x reduction) thus facilitating experimental scale up and improving practical utility. Because the cell culture area and input cell numbers are both known, surface seeding densities can be rapidly optimized to fit the needs of a particular tissue. The seeding module also allows co-cultures to be easily established by i) seeding one side of the culture region, ii) waiting for attachment, and iii) decoupling, flipping, and resealing the insert before directly seeding the other side. After a second phase of cell attachment, the membrane can be transferred directly to the culture module.

### Micro-perfusion and cell culture

Conventional culture platforms require syringe pumps to drive fluids through the platform. In an effort to make microfluidic assays accessible to a broad range of end users, we developed an option for a gravity fed module that provides micro-perfusion capabilities within our self-contained system. The micro-perfusion module can be used to maintain cell cultures or expose the apical or basolateral populations to experimental compounds. Each reservoir is individually addressable and can be used to establish physiological culture environments such as air-liquid interface culture, which is relevant in models for the cornea, skin, and lung. Our equipment-free perfusion option also makes the platform amenable for use in BSL3/4 facilities where syringe pumps are difficult to implement because of the risks of aerosol generation and issues arising from problematic decontamination [59]. Eqs 1 and 2 are used to estimate flow rates, required reservoir volumes, and shear stresses. Changing physical design parameters (channel heights and pressure drops) can easily be modified to achieve the desired perfusion characteristics. As shown in supplemental S5 Fig the modular approach also enables syringe pumps or other pumping mechanisms to be directly interfaced to provide shear stimulation if desired. In this demonstration, microfluidic channels were molded from PDMS and used to seal the channels against the culture insert under magnetic compression and provide a compartmentalized culture environment. PDMS has several favorable properties including optical clarity and ease of fabrication, but the material also has well known issues related to the partitioning of small hydrophobic molecules into the bulk [60,61]. In situations such as small molecule drug candidate testing, polyurethane can be used to minimize partitioning effects [62].

### Module prototyping and next generation systems

The laser cutting and lamination based fabrication process used here is ideal for design iterations and prototype development; with experience, a batch or 5–10 modules can be cut, aligned, and assembled in less than five minutes. Once a design has been finalized, modules can be scaled up for small batch production using hot embossing techniques or translated to injection molding for larger scale manufacturing. An inventory of mix-and-match modules to support experimental studies can established at either the lab prototype and advanced prototype stage. End users can thus easily introduce capabilities as needed.

The magnetic coupling technique used throughout also provides a simple approach to connect different tissue modules together (e.g. heart to lung) through a magnetic manifold to explore multi-module drug or toxin interactions (S5 Fig). Experimental scale up can be achieved by connecting multiple modules to a common perfusion source. The modular approach also enables in-line microfluidic immunoassays to be integrated with SEAM to analyze secreted proteins from small cell populations [63–65] and the layer-by-layer fabrication technique can support electrode integration to provide barrier integrity measurements in real time. The reversible SEAM architecture provides a technique to explore multi-tiered readouts from cells cultured on a variety of substrates with defined biochemical and biomechanical properties. The HA/PA material used in our work has been shown to inflate response to differential pressure across the interface [23]; this capability can be used in future studies to introduce organ-on-a-chip type mechanical stretch to tissue interfaces maintained within SEAM. With the recent advances in multi-material additive manufacturing, direct 3D printing can step beyond simple rigid housing materials and develop integrated rigid and flexible elements that can be used to reversibly seal a general class of cell culture membranes, and introduce mechanical stimulation to better mimic physiological microenvironments without compromising ease of use.

## Conclusions

The key objectives of the SEAM platform were to create i) a simple but functional architecture to explore cell interactions to external stimuli, ii) extend experimental capabilities by incorporating physiologically relevant culture interfaces, iii) streamline the cell introduction process and iv) simplify experimental workflows including imaging and nucleic acid analysis. Taken as a whole, the easy to use magnetically sealed modules and SEAM workflow could help to make compartmentalized microfluidic approaches more accessible to the scientific community and contribute to advances in barrier tissue research.

## Supporting Information

S1 Fig a) Chemical structure of peptide amphiphile with 16 carbon alkyl tail and A_3_V_3_K_3_ peptide sequence as designed by Stupp et al. b) Chemical structure of hyaluronic acid used in the supramolecular self-assembly process to make suspended membranes.
(TIF)

S2 Fig a) Representative AFM scan used to determine thickness of the self-assembled membrane. b) Representative AFM characterization curve to determine the Young’s modulus of the self-assembled membrane. C) Molecular weight cutoff (90% retention in donor channel) of membrane determined by quantitating transport of fluorescent dextran across the membrane from a donor to acceptor channel over 24 hours. Data represented by mean and standard deviation.
(TIF)

S3 Fig Forward and reverse primers for rat neuron gene expression study. (TIF)

S4 Fig a) Bioanalyzer RNA integrity number for off-chip RNA extraction using in channel lysis (C1 –C3, avg RIN = 9.5) and culture insert transfer method (L1 –L3, avg RIN = 9.96). A RIN value 10 represents intact RNA. b) RNA quantity from on-chip lysis (17.3 ± 1.17 ng) and collection compared with culture insert transfer method (34.1 ± 1.38 ng). Data represented as mean and standard error. * p<0.05.
(TIF)

S5 Fig Culture module connected to syringe pump and a second culture module via tubing. (TIF)

S1 Video Magnetic latching of SEAM module and attachment of perfusion module. (MOV)
