# Supplementary figures and images for: A Reversibly Sealed, Easy Access, Modular (SEAM) Microfluidic Architecture to Establish In Vitro Tissue Interfaces
Source: PLoS One. 2016 May 26;11(5):e0156341. doi: 10.1371/journal.pone.0156341 (PMC4881956; doi:10.1371/journal.pone.0156341)

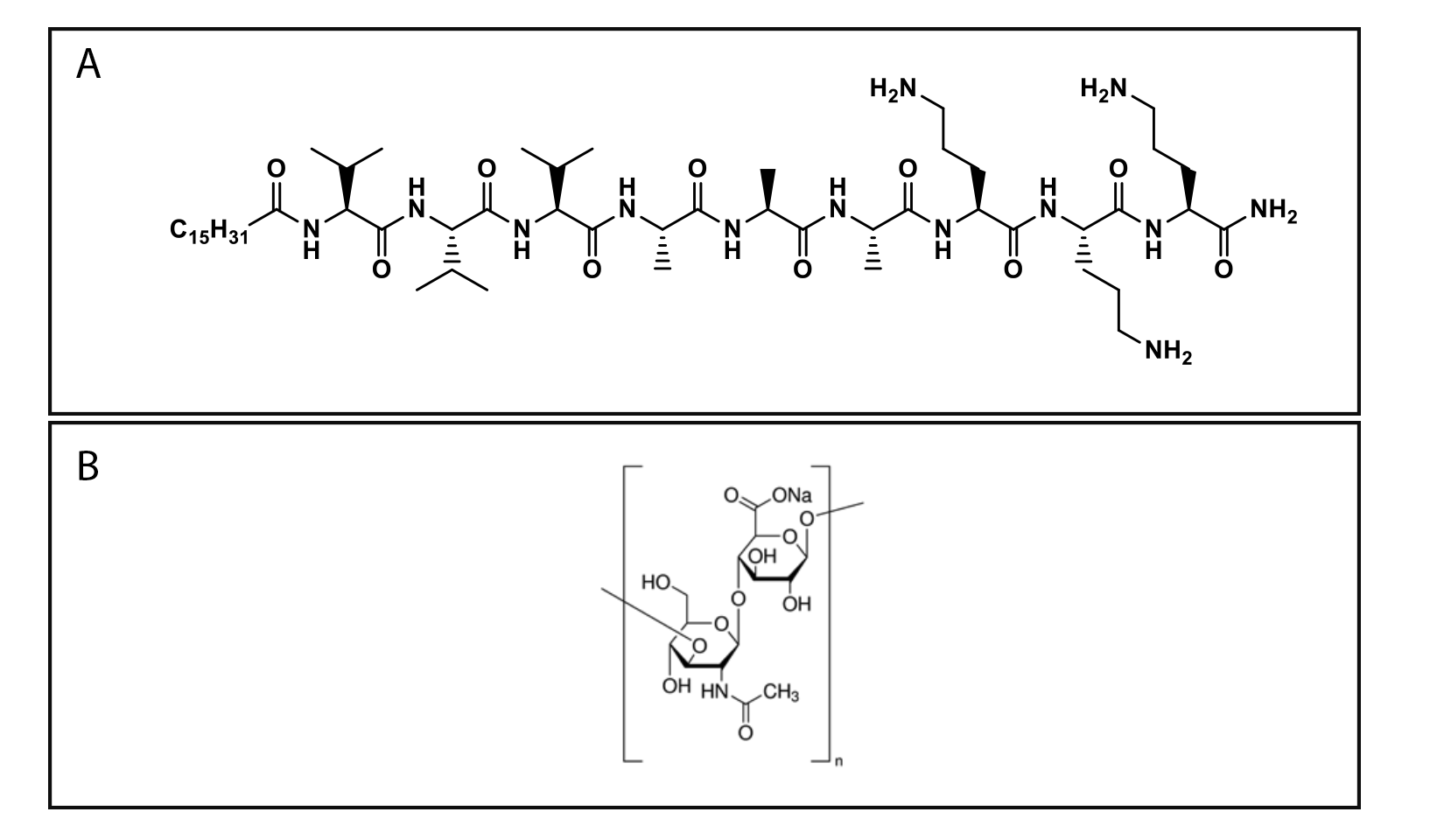

Supplement: S1 Fig — a) Chemical structure of peptide amphiphile with 16 carbon alkyl tail and A3V3K3 peptide sequence as designed by Stupp et al. b) Chemical structure of hyaluronic acid used in the supramolecular self-assembly process to make suspended membranes. (TIF) [file pone.0156341.s001.tif]

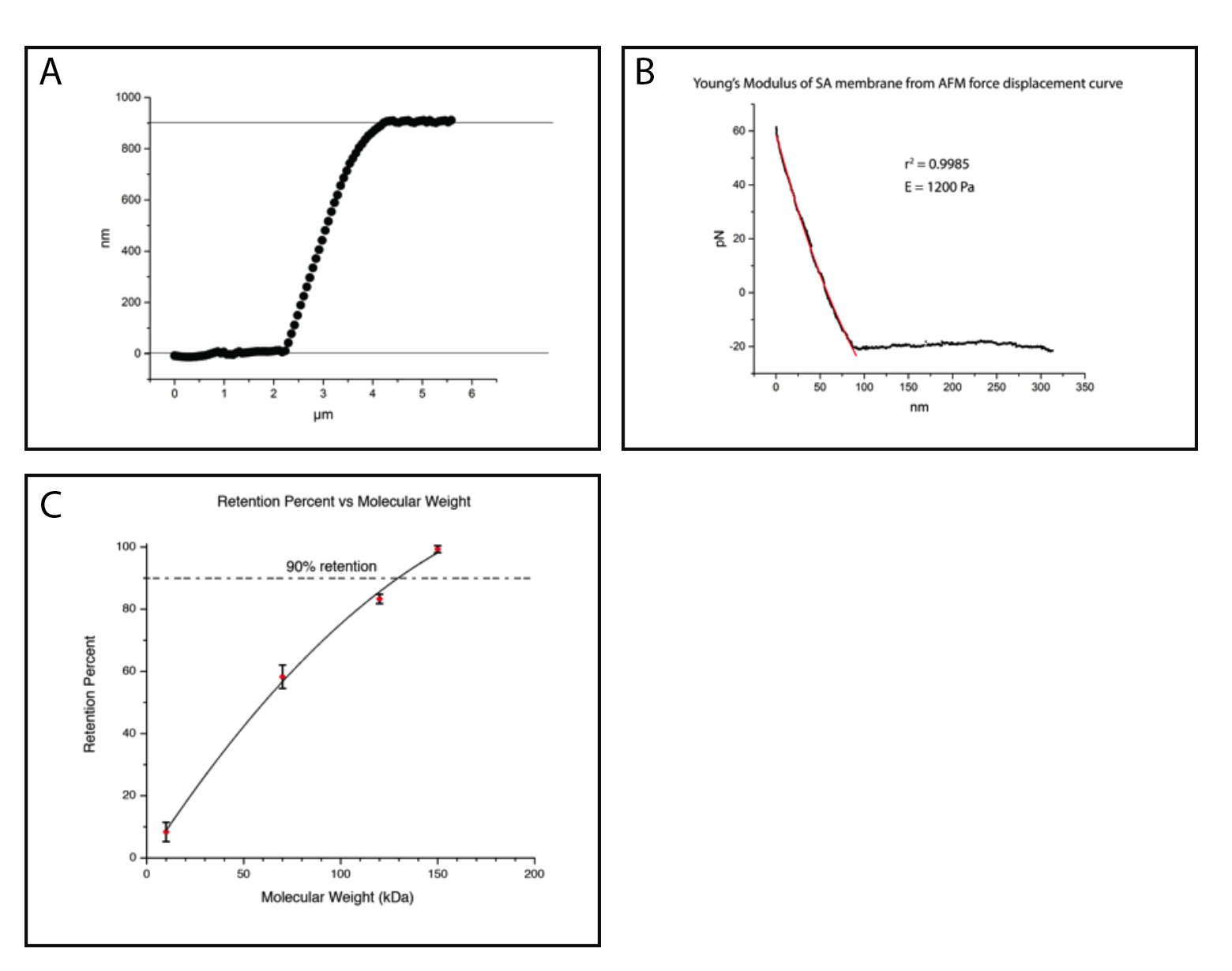

Supplement: S2 Fig — a) Representative AFM scan used to determine thickness of the self-assembled membrane. b) Representative AFM characterization curve to determine the Young’s modulus of the self-assembled membrane. C) Molecular weight cutoff (90% retention in donor channel) of membrane determined by quantitating transport of fluorescent dextran across the membrane from a donor to acceptor channel over 24 hours. Data represented by mean and standard deviation. (TIF) [file pone.0156341.s002.tif]

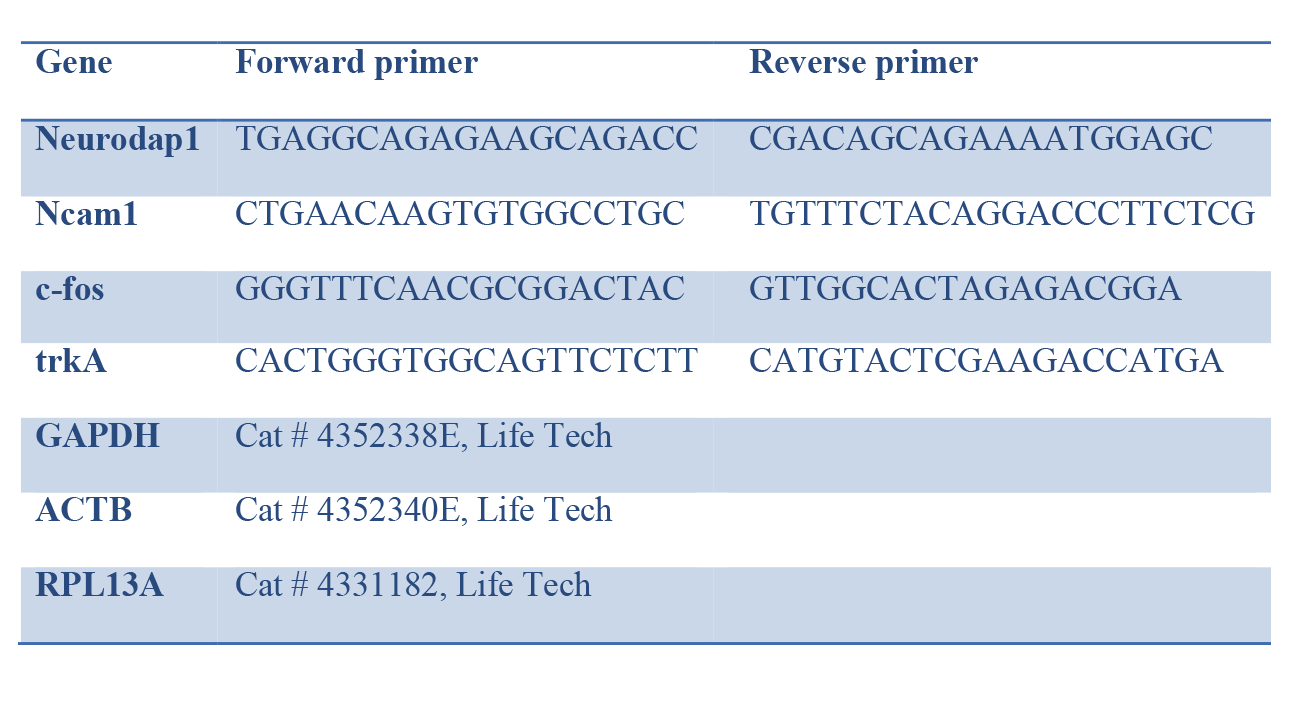

Supplement: S3 Fig — (TIF) [file pone.0156341.s003.tif]

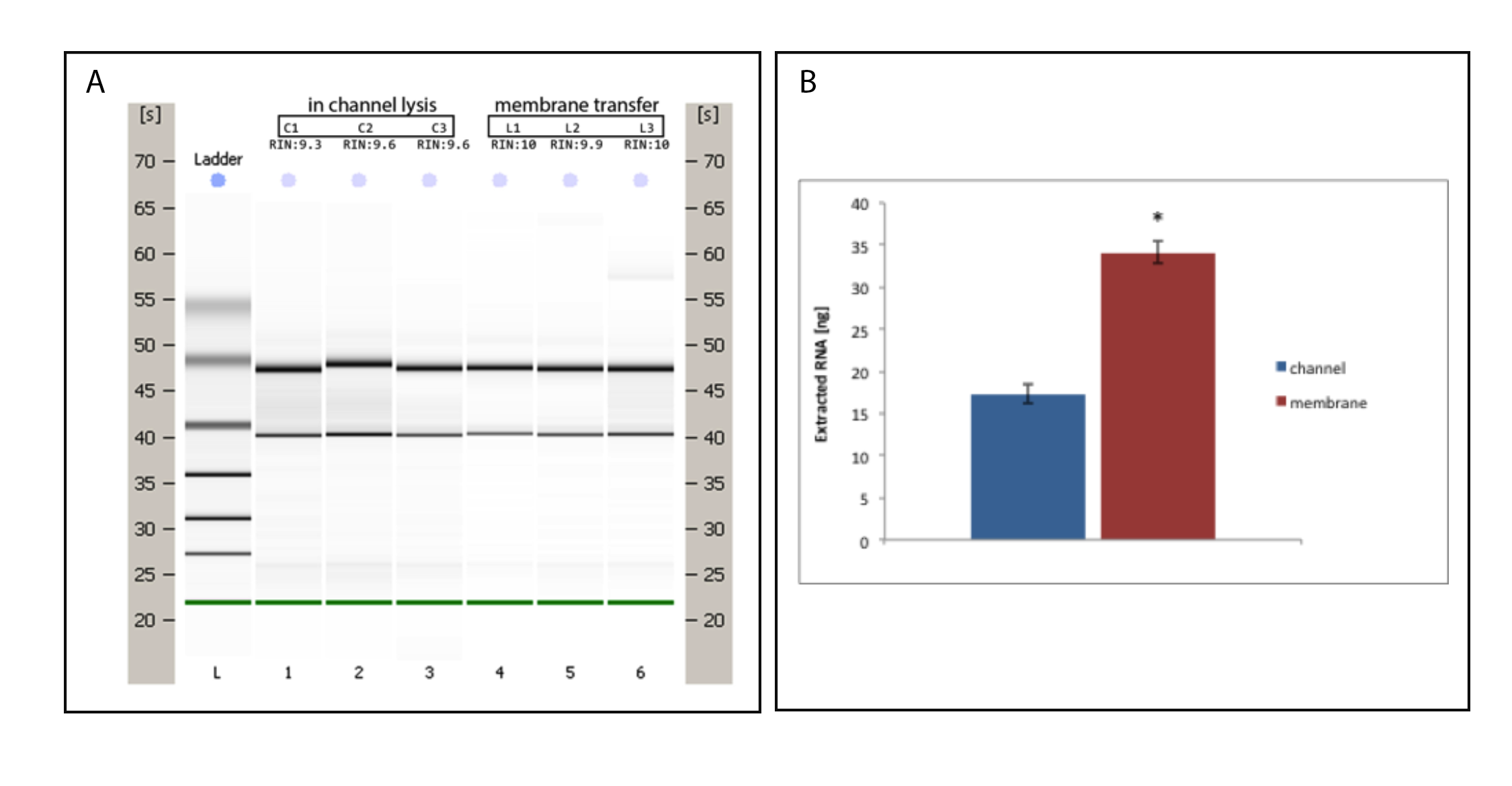

Supplement: S4 Fig — a) Bioanalyzer RNA integrity number for off-chip RNA extraction using in channel lysis (C1 –C3, avg RIN = 9.5) and culture insert transfer method (L1 –L3, avg RIN = 9.96). A RIN value 10 represents intact RNA. b) RNA quantity from on-chip lysis (17.3 ± 1.17 ng) and collection compared with culture insert transfer method (34.1 ± 1.38 ng). Data represented as mean and standard error. * p<0.05. (TIF) [file pone.0156341.s004.tif]

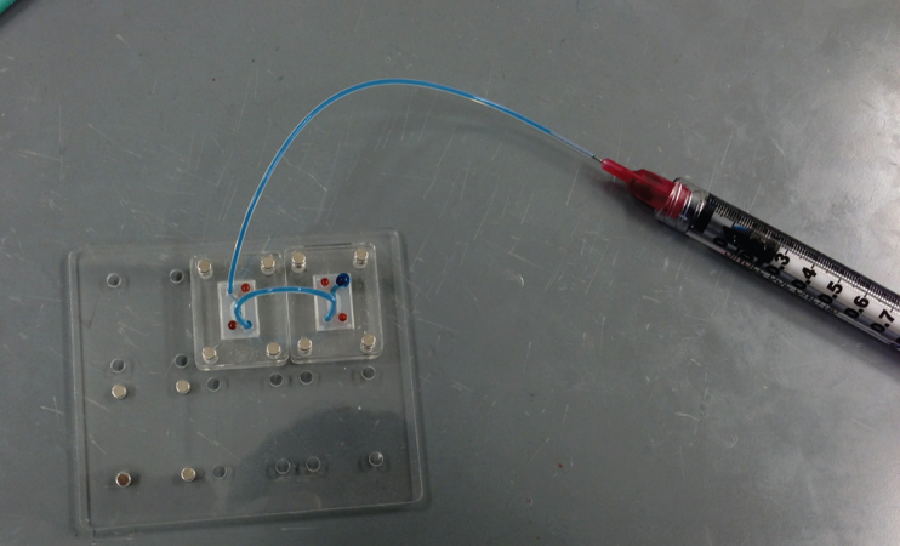

Supplement: S5 Fig — (TIF) [file pone.0156341.s005.tif]
